# Molecular Diffusion through Cyanobacterial Septal Junctions

**DOI:** 10.1128/mBio.01756-16

**Published:** 2017-01-03

**Authors:** Mercedes Nieves-Morión, Conrad W. Mullineaux, Enrique Flores

**Affiliations:** a Instituto de Bioquímica Vegetal y Fotosíntesis, Consejo Superior de Investigaciones Cientíﬁcas and Universidad de Sevilla, Seville, Spain; b School of Biological and Chemical Sciences, Queen Mary University of London, London, United Kingdom; Oregon State University

## Abstract

Heterocyst-forming cyanobacteria grow as filaments in which intercellular molecular exchange takes place. During the differentiation of N_2_-fixing heterocysts, regulators are transferred between cells. In the diazotrophic filament, vegetative cells that fix CO_2_ through oxygenic photosynthesis provide the heterocysts with reduced carbon and heterocysts provide the vegetative cells with fixed nitrogen. Intercellular molecular transfer has been traced with fluorescent markers, including calcein, 5-carboxyfluorescein, and the sucrose analogue esculin, which are observed to move down their concentration gradient. In this work, we used fluorescence recovery after photobleaching (FRAP) assays in the model heterocyst-forming cyanobacterium *Anabaena* sp. strain PCC 7120 to measure the temperature dependence of intercellular transfer of fluorescent markers. We find that the transfer rate constants are directly proportional to the absolute temperature. This indicates that the “septal junctions” (formerly known as “microplasmodesmata”) linking the cells in the filament allow molecular exchange by simple diffusion, without any activated intermediate state. This constitutes a novel mechanism for molecular transfer across the bacterial cytoplasmic membrane, in addition to previously characterized mechanisms for active transport and facilitated diffusion. Cyanobacterial septal junctions are functionally analogous to the gap junctions of metazoans.

## IMPORTANCE

Although bacteria are frequently considered just as unicellular organisms, there are bacteria that behave as true multicellular organisms. The heterocyst-forming cyanobacteria grow as filaments in which cells communicate. Intercellular molecular exchange is thought to be mediated by septal junctions. Here, we show that intercellular transfer of fluorescent markers in the cyanobacterial filament has the physical properties of simple diffusion. Thus, cyanobacterial septal junctions are functionally analogous to metazoan gap junctions, although their molecular components appear unrelated. Like metazoan gap junctions, the septal junctions of cyanobacteria allow the rapid intercellular exchange of small molecules, without stringent selectivity. Our finding expands the repertoire of mechanisms for molecular transfer across the plasma membrane in prokaryotes.

## OBSERVATION

Heterocyst-forming cyanobacteria grow as chains of cells—filaments—that can be hundreds of cells long, and the cells in the filament communicate with each other. Under deprivation of combined nitrogen, two cell types are found in the filament: vegetative cells that fix CO_2_ through oxygenic photosynthesis and heterocysts that fix N_2_ (1, 2). The process of heterocyst differentiation from vegetative cells involves intercellular molecular exchange, since differentiating heterocysts inhibit the differentiation of nearby cells (3), and this phenomenon involves the intercellular transfer of a regulator, such as the PatS morphogen (4, 5). In the diazotrophic cyanobacterial filament, heterocysts represent a low percentage of cells (about 5 to 10% in the model heterocyst-forming cyanobacterium *Anabaena* sp. strain PCC 7120 [hereinafter *Anabaena*]). For the filament to grow diazotrophically, an intercellular exchange of nutrients is necessary. The vegetative cells provide the heterocysts with photosynthate, mainly in the form of sucrose (see reference 6 and references therein), and the heterocysts provide the vegetative cells with fixed nitrogen, mainly in the form of glutamine and β-aspartyl-arginine dipeptide (see reference 7 and references therein). Because one heterocyst feeds about 5 to 10 vegetative cells with nitrogen, intercellular molecular exchange must also occur between vegetative cells. Indeed, as evidenced by the use of fluorescent markers, including calcein (8), 5-carboxyfluorescein (5-CF) (9), and the sucrose analogue esculin (6), intercellular molecular exchange takes place between the vegetative cells not only of diazotrophic filaments but also of filaments grown in the presence of combined nitrogen.

Intercellular molecular transfer appears to take place through conduits joining adjacent cells in the filament. Structures previously known as “microplasmodesmata” and now as “septal junctions” (10–12) may provide such conduits (8, 9). Proteins that are likely components of septal junctions have been identified. SepJ (13; also known as FraG [14]), FraC, and FraD (9, 15, 16) are integral membrane proteins located at the cell poles in the intercellular septa (13, 16). The integral membrane section of SepJ is homologous to permeases of the drug and metabolite exporter (DME) family (13). SepJ and FraD also have long extramembrane domains that, as discussed previously (12), may reside in the periplasmic area of the intercellular septa. These proteins are necessary for the formation of septal peptidoglycan nanopores (6), through which septal junctions appear to traverse the septal peptidoglycan (17). Structures observed by electron tomography of *Anabaena* that have been termed “channels” (18, 19) likely correspond to the nanopores.

Fluorescence recovery after photobleaching (FRAP) experiments with several fluorescent markers demonstrate the movement of molecules from cytoplasm to cytoplasm (6, 8, 9, 16). Net movement always occurs down the concentration gradient, and the kinetics appear to be independent of direction. For example, the rate constant for movement of the sucrose analog esculin from vegetative cells to heterocysts is similar to the rate constant for the reverse movement from heterocysts to vegetative cells (6). The fact that a range of dyes can be exchanged on rapid timescales, including fluorescein derivatives with no resemblance to any known *Anabaena* metabolite, suggests that the mechanism has low selectivity. Nonetheless, the participation of proteins (with homology to permeases in the case of SepJ) in the process of intercellular transfer led us to inquire further into its physical nature. Temperature dependence provides a way to distinguish simple diffusion from carrier-mediated facilitated diffusion. In a facilitated diffusion mechanism, the substrate interacts strongly with the membrane transporter protein, forming a bound intermediate state. Such a mechanism confers specificity but also introduces an activation energy barrier. This makes the transport strongly temperature dependent, with a *Q*_10_ (the factor by which the rate increases with a 10°C increase in temperature) typically of at least 2 (20, 21). In contrast, the rate of simple diffusion will be proportional to the absolute temperature, giving a *Q*_10_ of about 1.035 in the physiological temperature range. Here, we report the observation that intercellular transfer of the fluorescent markers in *Anabaena* exhibits a dependence on temperature that denotes a process of simple diffusion.

### Temperature dependence.

**FIG 1:**
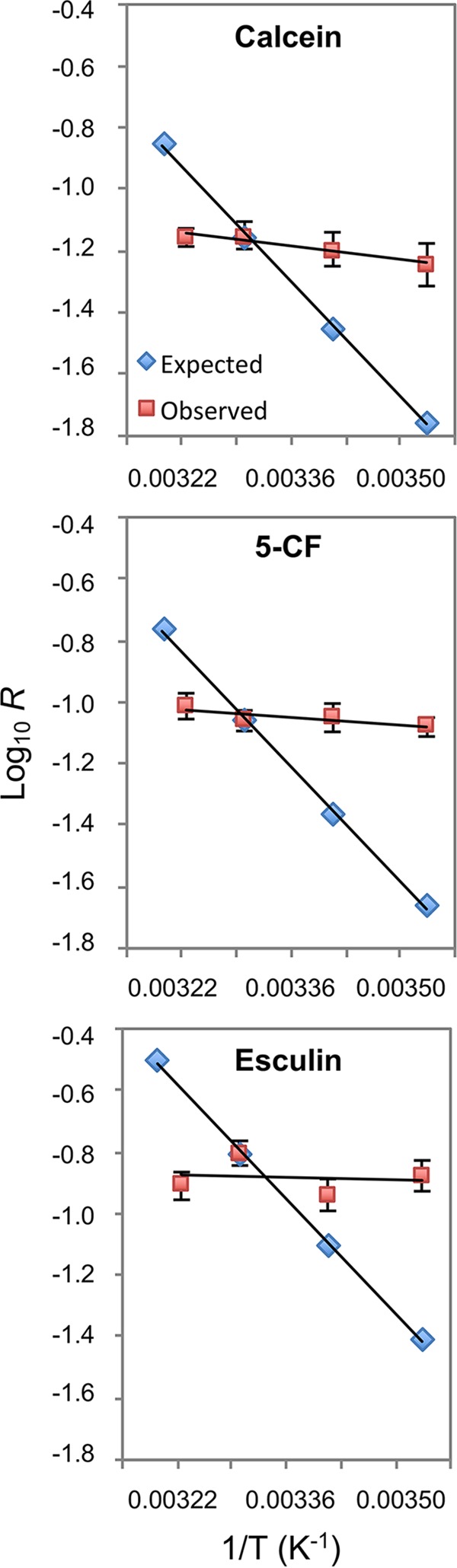
Effect of temperature on the intercellular transfer of calcein, 5-CF, and esculin in *Anabaena* (Arrhenius plots). BG11-grown filaments were used in FRAP assays as described in “Methods.” The assay temperature was set at 10, 20, 30, or 37°C. Observed, experimental data (mean and standard deviation of 26 to 43 filaments subjected to FRAP analysis for each marker and temperature, except that 43 to 126 filaments were tested at 30°C); Expected, values calculated assuming a *Q*_10_ of 2, taking as reference the experimental value at 30°C. FRAP data are presented as the recovery rate constant, *R* (s^−1^).

Intercellular molecular transfer was investigated with the fluorescent markers calcein, 5-CF, and esculin. Calcein and 5-CF are loaded into the cells as hydrophobic acetoxymethyl esters, which are hydrolyzed by cytoplasmic esterases, producing hydrophilic, fluorescent compounds that are stably retained within the cells (8, 9). Esculin is taken up into the *Anabaena* cells by permeases that build up an intercellular pool appropriate for analysis (6). FRAP assays were carried out at different temperatures, from 10 to 37°C, in *Anabaena* filaments that had been grown at 30°C. The constant, *R*, of the rate of fluorescence recovery in the bleached cells was calculated (9). Figure 1 (red squares) shows the results of the FRAP analysis with the three markers. We found *Q*_10_ values of 1.078, 1.045, and 1.015 for calcein, 5-CF, and esculin, respectively. For comparison, the expected values calculated assuming a *Q*_10_ of 2 (taking as reference the experimental value at 30°C) are also shown for the three markers (Fig. 1, blue rhombi). The observed *Q*_10_ values are fully consistent with a mechanism of simple diffusion but not with active transport or carrier-mediated facilitated diffusion.

### Concluding remarks.

Cells in the filaments of heterocyst-forming cyanobacteria are joined by septal junctions that functionally resemble metazoan gap junctions (10–12, 17). The observation that intercellular molecular transfer in *Anabaena* is proportional to the absolute temperature, presented here, strongly supports that septal junctions allow simple diffusion of small molecules, as gap junctions do (22, 23). The proteins that are putative components of septal junctions bear no homology, however, with connexins, which are the constituents of the connections that form the gap junctions (24). In gap junctions, the plasma membranes of the two cells are in close proximity. It should be noted that linking two cells in a cyanobacterial filament requires a larger and more complex structure, since the intercellular channel must span the periplasm and the intervening peptidoglycan layer. A major task for future research is to explore whether septal junctions can, like gap junctions (22, 23), be regulated by gating.

### Methods.

*Anabaena* sp. strain PCC 7120 was grown in BG11 medium modified to contain ferric citrate instead of ferric ammonium citrate (25) at 30°C in the light (ca. 25 to 30 μmol photons m^−2^ s^−1^) in shaken (100 rpm) liquid cultures. For esculin transfer assays, filaments from 1 ml of culture were harvested, resuspended in 500 µl of fresh growth medium, mixed with 15 µl of saturated (~5 mM) aqueous esculin hydrate solution (Sigma-Aldrich), and incubated for 1 h in the dark with gentle shaking at 30°C prior to washing 3× in growth medium, followed by dark incubation for 15 min in 1 ml medium at 30°C with gentle shaking. Cells were then washed, concentrated about 20×, and spotted onto BG11 medium solidified with 1% (wt/vol) Difco Bacto agar. Small blocks of agar with cells adsorbed on the surface were placed in custom-built, temperature-controlled sample holders under glass coverslips and at the different assay temperatures. Cells were visualized with a laser-scanning confocal microscope (Leica TCS SP5) using a 63× oil immersion objective (1.4 numerical aperture [NA]). Fluorescence was excited at 355 nm, with detection of esculin at 443 to 490 nm. High-resolution imaging used a 6× line average with an optical section of ~0.7 µm. FRAP measurements were performed without line averaging and with a wide pinhole that gave an optical section of ~4 µm. After capturing a prebleach image, the fluorescence of a defined region of interest was bleached out by scanning this region at ~6-times-higher laser intensity, and the recovery was then recorded in a sequence of full-frame images.

For calcein and 5-CF transfer assays, calcein and 5-CF staining and FRAP analysis were performed as previously reported (8, 9). Cell suspensions were spotted onto agar and placed in a custom-built, temperature-controlled sample holder as described above, and measurements were carried out at the temperatures indicated in the legend to Fig. 1. For both calcein and 5-CF, cells were imaged with a Leica HCX Plan-Apo 63×, 1.4 NA oil immersion objective attached to a Leica TCS SP5 confocal laser-scanning microscope with a 488-nm line argon laser as the excitation source. Fluorescence emission was monitored by collection across windows of 500 to 525 nm with a 150-μm pinhole. After an initial image was recorded, bleaching was carried out by an automated FRAP routine which switched the microscope to X-scanning mode, increased the laser intensity by a factor of 10, and scanned a line across 1 cell for 0.137 s before reducing the laser intensity, switching back to XY-imaging mode and recording a sequence of images typically at 1-s intervals.

For FRAP data analysis, the kinetics of fluorescent marker transfer between vegetative cells located in the middle of filaments (hence, with 2 cell junctions) was quantified by measuring the recovery rate constant *R* from the formula C_B_ = C_0_ + C_R_ (1 − e^−2*R*t^), where C_B_ is fluorescence in the bleached cell, C_0_ is fluorescence immediately after the bleach and tending towards (C_0_ + C_R_) after fluorescence recovery, t is time, and *R* is the recovery rate constant due to transfer of the marker from one neighbor cell (9).
